# Alcohol Septal Ablation Before TAVR in Patients With Concomitant LVOT Obstruction and Severe Aortic Stenosis: A Single-Center Study

**DOI:** 10.1016/j.jscai.2025.104005

**Published:** 2025-10-15

**Authors:** Gerardo V. Lo Russo, Mohamad A. Alkhouli, Mayra E. Guerrero, Charanjit S. Rihal, Mackram F. Eleid

**Affiliations:** a Department of Cardiovascular Medicine, Mayo Clinic, Rochester, Minnesota; b Department of Clinical Sciences and Community Health, Cardiovascular Section, University of Milan, Milan, Italy; c Department of Clinical and Interventional Cardiology, GMV Care & Research, Maria Cecilia Hospital, Cotignola, Italy

**Keywords:** alcohol septal ablation, aortic stenosis, hypertrophic cardiomyopathy, left ventricular outflow tract obstruction, transcatheter aortic valve replacement

Left ventricular outflow tract obstruction (LVOTO) and aortic stenosis (AS) are 2 distinct conditions associated with increased left ventricular afterload. The LVOTO is a dynamic phenomenon, highly dependent on ventricular loading. The sudden reduction in afterload after AS treatment can exacerbate LVOTO, potentially leading to catastrophic outcomes.^1^ The LVOTO after transcatheter aortic valve replacement (TAVR) is a rare but serious complication, reported in 1.8% to 13.3% of patients, with higher incidence among those with asymmetric septal hypertrophy, the presence of systolic anterior motion, or pre-existing elevated LVOT gradients.^2^ When both LVOTO and AS are diagnosed, careful evaluation and therapeutic planning are essential.^3^

Septal radiofrequency ablation or electrosurgical myotomy may effectively treat LVOTO but remain underexplored in patients with AS.^4^ A stepwise approach involving alcohol septal ablation (ASA) followed by TAVR has been proposed for these patients.^5^^,^^6^ However, data on the efficacy and safety of combining these procedures are limited.

We hypothesized that ASA can effectively reduce LVOTO in patients with concomitant AS and prevent adverse hemodynamic consequences after TAVR. The aims of this study are to evaluate the safety and efficacy of ASA in patients with concomitant LVOTO and severe AS and to assess the effect of ASA in patients undergoing TAVR.

We conducted a single-center, retrospective cohort study of patients with LVOTO and severe AS undergoing ASA before TAVR at Mayo Clinic (May 2018 to June 2024). The LVOTO was defined as a peak LVOT gradient ≥50 mm Hg. Severe AS was defined as an aortic valve area ≤1.0 cm^2^, mean gradient ≥40 mm Hg, or peak velocity ≥4.0 m/s. The primary end point was ASA efficacy, defined as a postprocedural LVOT peak gradient <50 mm Hg. Secondary end points included procedural success of TAVR, periprocedural complications, and 30-day mortality after TAVR.

Ten patients underwent ASA prior to TAVR. The median age was 83 years (IQR, 79-85), and 5 (50%) were women. The mean pre-ASA peak LVOT gradient was 89.8 ± 37.3 mm Hg, decreasing to 17.9 ± 16.1 mm Hg post-ASA (*P* < .001) (Table 1). Systolic anterior motion of the mitral valve was present in 6 patients pre-ASA and resolved in 2 post procedure. ASA was well tolerated, with no major complications, and 3 (30%) patients required permanent pacemaker implantation post-ASA. The TAVR with a balloon-expandable valve was performed in all patients at a median of 59 days post-ASA. Periprocedural complications occurred in 3 (30%) patients, including 2 cases of cardiac tamponade and 1 pacemaker implantation. Both cases of tamponade occurred in patients with an annular area of 6.10 cm^2^ and elevated aortic valve calcium score (3699 and 5497, respectively) treated with a 29-mm SAPIEN 3 valve (Edwards Lifesciences). One patient did not survive, while the other underwent successful emergent surgical repair (30 day survival after TAVR was 90% [9/10]).

**Table 1:** Procedural characteristics and outcomes.

Patient	Age, y	Septal ablated	Amount of ethanol injected, mL	Septal thickness pre ASA, mm	LVOT peak gradient^a^ pre-post ASA, mm Hg	SAM pre-post	Post-ASA PM implant	AV mean gradient, mm Hg	CT annulus area, cm^2^	SAPIEN 3 size, mm	ASA to TAVR, d	Complication following TAVR	Alive at 30 d
1	81	1°	1.5	17	90-13	0-0	No	49	2.40	23	55	None	Yes
2	79	1°	1.0	15	88-47	0-0	No	52	3.50	23	132	None	Yes
3	84	1°	1.2	23	59-13	1-1	No	42	6.10	29	49	Tamponade	No
4	84	2°	1.5	15	68-4	0-0	No	40	3.90	23	81	PM	Yes
5	69	1°	1.5	21	50-43	1-1	No	25	4.30	23	114	None	Yes
6	85	2°	1.0	23	150-27	1-0	No	54	5.10	26	63	None	Yes
7	87	1°	1.0	19	100-4	1-0	Yes	35	5.70	26	280	None	Yes
8	77	1°	1.0	19	59-5	0-0	Yes	52	3.00	20	30	None	Yes
9	82	2°	1.0	19	76-18	1-1	Yes	57	6.10	29	34	Tamponade	Yes
10	85	1°	1.0	17	158-5	1-1	No	48	4.16	23	50	None	Yes

ASA, alcohol septal ablation; AV, aortic valve; LVOT, left ventricular outflow tract; PM, pacemaker; SAM, systolic anterior motion; TAVR, transcatheter aortic valve replacement.

a LVOT gradient was obtained by echocardiography before ASA and within one month following the procedure.

The findings of this study indicate that ASA effectively reduces LVOTO in patients with severe AS, facilitating successful TAVR. The lack of a control group of severe AS patients with LVOTO who did not undergo intervention is an important limitation. The optimal interval between ASA and TAVR remains unclear, but a longer delay may allow for improved tissue remodeling. Annular injury was more common than expected with TAVR in this population, possibly due to the localized necrosis induced by ASA and the associated inflammation. Strategies to mitigate complications during TAVR in this high-risk population may include avoiding excessive annular oversizing and considering use of self-expanding valves.

In summary, ASA can be safely performed in patients with severe AS and LVOTO, mitigating the risk of hemodynamic decompensation after TAVR. This study is hypothesis-generating, and further studies in larger populations are warranted to clarify strategies to optimize outcomes in this challenging cohort.
